# American Journal of Insanity

**Published:** 1845-02

**Authors:** 


### THE MEDICAL EXAMINER.

PHILADELPHIA, FEBRUARY, 1845.

### AMERICAN JOURNAL OF INSANITY.

In acknowledging the receipt of the number for January of the present year, (Vol. 1, No. 3,) of this excellent periodical, we embrace the occasion to say a few words in regard to its character and objects.

The *title* of the Journal presents little attraction for the mass of readers, and but for the benevolent objects contemplated by its publication few perhaps would care to look into its pages; but those who do will find, that it embraces a variety of topics of the deepest interest, to the lay as well as the professional; and, in most instances, discussed with such clearness and ability as to command the attention and respect of the most desultory inquirer.

In our "*Record*" we have inserted some extracts, from which our readers will discover something of the tenor of the work, and it is our purpose to glean largely from its pages on future occasions, and thus aid in disseminating some of the important information with which it is abundantly stored. It is "*edited by the Officers of the New York*

*State Lunatic Asylum, Utica*," and the object of its publication "is to popularize the study of insanity,—to acquaint the general reader with the nature and varieties of this disease, methods of prevention and cure. "We also hope," say the Editors, "to make it useful and interesting to members of the medical and legal profession, and to all those engaged in the study of the phenomena of mind."

